# Cockpit voice recorder transcript data: Capturing safety voice and safety listening during historic aviation accidents

**DOI:** 10.1016/j.dib.2021.107602

**Published:** 2021-11-20

**Authors:** Mark C. Noort, Tom W. Reader, Alex Gillespie

**Affiliations:** a Leiden University, Law School, the Netherland; b London School of Economics and Political Science, United Kingdom; c Bjørknes University College, Norway

**Keywords:** CRM, Crew Resource Management, Cockpit voice recorders, Safety voice, Safety listening, Aviation accidents, CRM

## Abstract

Cockpit Voice Recorder (CVR) transcripts capture audio data within cockpit environments. This aids the investigation of causal factors contributing to aviation accidents by revealing communication and other sounds prior to aviation accidents. This dataset contains 172 unique CVR transcripts (with 21,626 lines of transcript: averaging: 106.001 conversational turns; SD = 51.727, range: 1-641), and capturing approximately 15% of historic aviation fatalities in commercial and corporate aviation between 1962 and 2018. CVR transcripts involved airlines registered across 42 countries, with accidents occurring across 50 countries. The dataset was compiled by extracting CVR transcripts from three primary data sources and excluding duplicate and non-English entries. The data contains variables describing the (i) raw data, (ii) content and characteristics of the CVR transcripts, and (iii) behaviours coded by research assistants in support of the associated research article. The data existed of conversational turns amongst flight crew (total = 19,393; within transcripts: *m* = 112.750; SD = 124.829) and other data (*n* = 2213; within transcripts: *m* = 12.866; SD = 14.452; e.g., background sounds, transcriber notes). Conversational turns were uttered by junior (39.00%) and senior (35.44%) flight crew, and others (25.56%). The dataset enables future research through providing the first integrated dataset on communication behaviours prior to historic aviation accidents. Moreover, the dataset may support safety management through enabling the identification of communication behaviours contributing to accidents and the design of novel interventions. This data-in-brief is a co-submission associated with the research article: M. C. Noort, T.W. Reader, A. Gillespie. (2021). Safety voice and safety listening during aviation accidents: Cockpit voice recordings reveal that speaking-up to power is not enough. Safety Science.

## Specifications Table

**Table:** 

Subject	Social Sciences: Safety Research
Specific subject area	Safety voice and safety listening during historic accidents (1962-2018) in corporate and commercial aviation
Type of data	Table
How data were acquired	Extraction and integration of Cockpit voice recorder transcripts from 172 publicly available air crash investigation reports.
Data format	Raw Coded
Parameters for data collection	Cockpit Voice Recorder (CVR) transcripts were included when they were available on three online databases. Data collection was limited to accidents that occurred between 1962 and 2018 (i.e., the data range available at the time) in corporate and commercial aviation (i.e., general aviation accidents were not included). Reports without transcripts were not included. Duplicate entries and transcripts in languages other than English were removed.
Description of data collection	In January 2018, 372 transcripts from historic aviation accidents were identified across three online databases. Transcripts were manually downloaded and integrated into a single larger file containing all transcripts. Narrative summaries and available information that could aid interpretation was downloaded alongside the cockpit voice recorder transcripts. Data was coded with developed coding schemes for descriptive information about the transcript and conversational turn, speaker, and content (e.g., safety voice, safety listening). The final dataset contained 172 transcripts after duplicate, irretrievable and non-English transcripts were removed.
Data source location	Primary data sources available on: 1. Tailstrike (2019). Cockpit voice recorder database. https://www.tailstrike.com/database.htm 2. Plane Crash Info (2019). Last Words. http://www.planecrashinfo.com/lastwords.htm 3. Aviation-Safety Network (2019). Transcripts. https://aviation-safety.net/investigation/cvr/transcripts/
Data accessibility	With the article
Related research article	[1] M.C. Noort, T.W. Reader, A. Gillespie. Safety voice and safety listening during aviation accidents: Cockpit voice recordings reveal that speaking-up to power is not enough. Safety Science 139 (2021) 105260. https://doi.org/10.1016/j.ssci.2021.105260

## Value of the Data

• The CVR transcript dataset is important because it is the only available dataset of its kind containing communication prior to historic aviation accidents (i.e., an out-dated set is no-longer available [3]). Moreover, unlike the majority of research on safety voice and safety listening [2], it captures these behaviours in emergency scenarios posing actual levels of fatal risk.
• The dataset may benefit safety practitioners and researchers that are interested in utilising the data to identify, conceptualise and/or mitigate patterns in communication behaviours that may contribute to accidents. This is consistent with research using small numbers of CVR transcript that indicated transcripts can reveal in-situ interactions between safety-critical staff [4]. For instance, by identifying factors that impact on communication scholars may improve and design training programs such as Crew Resource Management [5,6].
• The dataset may be used to enhance safety management theory through analysing the nature of safety voice speech [7] or providing additional coding of communication patterns present in the data.
• Finally, by supplementing the data with data from ‘routine’ or ‘normal flights the dataset would support designs aiming to clarify the relationship between communication and the prevention of accidents.

## 1 Data Description

Cockpit voice recorders are technical equipment installed with the intention to capture conversations and sounds on the flight deck and enable accident analyses [8]. The dataset contains 172 cockpit voice recorder transcripts integrated into a single file (provided as .sav and .csv). Each row represents a conversational turn (i.e., any words uttered by a speaker until another person speaks). Each column represents a variable that was extracted from the original data sources. This datafile includes three types of variables. First, case variables describe high level information about the accident (e.g., case identifier, original data source, date, location of accident, number of people on board). Second, variables labelled ‘cvr’ describe the content and characteristics of the transcript (e.g., message spoken, role of person speaking, etc.). Third, variables labelled ‘coding’ capture variables that describe behaviours as coded by research assistants. Variables are described within the SPSS ‘labels’, and data values are labelled where appropriate. The coding framework used to code the data and the full variable list are provided as additional .csv files.

## 2 Experimental Design, Materials and Methods

Cockpit voice recorder transcripts were retrieved by January 2018 from accident investigation reports that were available on three online repositories (tailstrike.com, aviation-safety.net, planecrashinfo.com), written in English, and unique (i.e., duplicates were removed).

As highlighted in the accompanying article, the following data was extracted from the transcripts: “(i) flight number, (ii) date of incident, (iii) audio source, (iv) airline country registration, (v) incident airspace, (vi) flight phase, (vii) crew and passenger numbers, (viii) fatalities, (ix) damage, (x) attributed causal factors, (xi) transcript conversational turn, (xii) speaker. To provide interpretative context, narrative summaries and legends were included.”

In addition, the Cockpit voice recorder transcripts were coded manually by research assistants using legends available with identified transcripts, or using available coding schemes: “(i) turn number (i.e., sequential within transcripts), (ii) turn type (i.e., conversation, background sounds, notes/information), (iii) conversational turn (i.e., sequential for conversation turns within transcripts), (iv) person speaking (captain, first officer, flight engineer, flight crew with unclear role, cabin crew, air traffic control, other aircraft, ground operations, other), (v) the hazard raised (i.e., if one was raised, using the words of the conversational turn), vi) how others listened to the hazard raised (action, affirmed, disaffirmed, ignored, unclear), and (vii) the type of hazard based on [NATS’] air traffic control classification scheme (i.e., ATC interaction, Crew interaction, Distraction, Equipment/fuel, Location, Manoeuvring, Weather, Pilot actions, Planning, Company actions, Other/unclear)”. Research assistants were trained to digitally code complex variables (i.e., hazard raised, listening behaviour, type of hazard) in Microsoft Excel using the coding framework (detailing decision rules and examples) by coding training transcripts, comparing this to each other and the lead author, and discussing discrepancies.

Additional variables coded whether a conversational turn contained safety voice and an alternative online source for the accident investigation report.

## Ethics Statement

Ethical approval was obtained from the London School of Economics and political science's research ethics committee (#1051). Whilst data emerged from publicly available air crash investigation reports, speaker identity was anonymised by providing roles.

## CRediT authorship contribution statement

**Mark C. Noort:** Conceptualization, Methodology, Investigation, Formal analysis, Writing – original draft, Writing – review & editing, Visualization, Supervision. **Tom W. Reader:** Conceptualization, Validation. **Alex Gillespie:** Conceptualization, Validation.

## Declaration of Competing Interest

The authors have declared no competing interests.

## Appendix Supplementary materials

Image, application 1

Image, application 2

Image, application 3

Image, application 4
